# Ultrasonographic Evaluation of Botulinum Toxin Injection Site for the Medial Approach to Tibialis Posterior Muscle in Chronic Stroke Patients with Spastic Equinovarus Foot: An Observational Study

**DOI:** 10.3390/toxins9110375

**Published:** 2017-11-18

**Authors:** Alessandro Picelli, Alessio Baricich, Elena Chemello, Nicola Smania, Carlo Cisari, Marialuisa Gandolfi, Nicoletta Cinone, Maurizio Ranieri, Andrea Santamato

**Affiliations:** 1 Neuromotor and Cognitive Rehabilitation Research Center, Department of Neurosciences, Biomedicineand Movement Sciences, University of Verona, Verona 37134, Italy; alessandro.picelli@univr.it (A.P.); elenachemello13@gmail.com (E.C.); nicola.smania@univr.it (N.S.); marialuisa.gandolfi@univr.it (M.G.); 2 Health Sciences Department, Università del Piemonte Orientale, Novara 28100, Italy; alessio.baricich@med.uniupo.it (A.B.); carlo.cisari@med.uniupo.it (C.C.); 3 Physical Medicine and Rehabilitation Section, “OORR Hospital”, University of Foggia, Foggia 71122, Italy; n.cinone@gmail.com (N.C.); maurizio.ranieri@unifg.it (M.R.)

**Keywords:** muscle spasticity, rehabilitation, ultrasonography

## Abstract

The tibialis posterior muscle is a frequent target for injection of botulinum toxin during the management of spastic equinovarus foot in adults with post-stroke spasticity. Although it is deep-seated, the needle insertion into the tibialis posterior muscle is usually performed using anatomical landmarks and safety information obtained from healthy subjects and cadavers. Our aim was to evaluate the botulinum toxin injection site for the medial approach to the tibialis posterior muscle in chronic stroke patients with spastic equinovarus foot. Forty-six patients were evaluated at the affected middle lower leg medial surface with ultrasonography according to the following parameters: tibialis posterior muscle depth, thickness, and echo intensity. As to the spastic tibialis posterior, we found a mean muscle depth of 26.5 mm and a mean muscle thickness of 10.1 mm. Furthermore we observed a median tibialis posterior muscle echo intensity of 3.00 on the Heckmatt scale. The tibialis posterior muscle thickness was found to be inversely associated with its depth (*p* < 0.001) and echo intensity (*p* = 0.006). Furthermore, tibialis posterior muscle depth was found to be directly associated with its echo intensity (*p* = 0.004). Our findings may usefully inform manual needle placement into the tibialis posterior for the botulinum toxin treatment of spastic equinovarus foot in chronic stroke patients.

## 1. Introduction

Stroke is a main cause of adult disability [1]. Damage to the descending tracts and sensory-motor networks may lead to the upper motor neuron syndrome (UMNS) [2,3,4]. Spasticity is a positive feature of the UMNS that has been found to involve both the upper and lower limbs in 27.0% and the lower limb only in 7.1% of stroke patients at 6 months since onset [5]. It has been defined as follows: “a state of increased muscle tone with exaggerated reflexes characterized by a velocity-dependent increase in the resistance to passive movement” [6]. Spastic paresis due to the UMNS may interfere with motor function, leading to the need for clinical interventions, such as drugs administration, physical therapy or other rehabilitation procedures [7,8,9].

Botulinum toxin type A (BoNT-A) has been established as safe and effective for the reduction of focal spasticity [7]. In adults with post-stroke spasticity, the pattern most commonly treated with BoNT-A at the affected lower limb is the equinovarus/equinus foot [10], which is a condition that frequently complicates functional recovery, interferes with walking ability, and limits the activities of daily living [11]. The main muscles involved in the equinovarus foot pattern are the gastrocsoleus complex, and tibialis posterior (TP) muscle [12]. In particular, the TP muscle, which represents the main foot invertor and may assist in powerful plantar flexion, is frequently targeted for BoNT-A injection in order to treat the equinovarus deformity by reducing muscle hypertonia [11,13]. 

The major causes for the loss of BoNT-A response in adults with focal spasticity are the following: inaccurate muscle selection and identification for injection, insufficient drug dosages, inadequate injection technique, development of changes in the muscle, and formation of neutralizing antibodies [14]. Concerning the muscles involved in the equinovarus foot pattern [12], accuracy of needle insertion is crucial for the treatment of gastrocnemius muscle spasticity with BoNT-A, according to the currently available literature, which has reported that neither manual needle placement (MNP) nor electrical stimulation guidance are fully accurate in adult patients with spastic equinus after stroke [15,16,17]. 

As to the TP muscle, although it is deep-seated within the lower leg and not easily accessible for needle placement, the needle insertion into it is usually performed with the guidance of anatomical landmarks [13,14]. Previous research into TP muscle injection in adults has suggested safety windows for needle placement based on the evaluation of healthy volunteers and cadavers [13,18,19,20,21]. On these bases, and considering that MNP is actually the most commonly used BoNT-A injection technique for the treatment of spasticity in adults [22], there is a lack of information about the BoNT-A injection site for spastic TP muscle in adult patients. Thus, the aim of this study was to evaluate, by means of ultrasonography, the BoNT-A injection site for the medial approach to TP muscle in chronic stroke patients with spastic equinovarus foot.

## 2. Results

Forty-six chronic stroke patients were recruited from among 136 consecutive outpatients. The enrolment period was from October 2016 to May 2017. Table 1 presents the patients’ demographic and clinical features.

As to the correlation analysis, TP muscle thickness was found inversely associated with its depth (*p* < 0.001; ρ = −0.541) and echo intensity (*p* = 0.006; ρ = −0.403). Furthermore, TP muscle depth was found to be directly associated with its echo intensity (*p* = 0.004; ρ = 0.416). Table 2 reports the results of the Spearman rank correlation test.

## 3. Discussion

Consistent with the ultrasonographic evaluation of the affected middle lower leg medial surface performed on a sample of adult chronic stroke patients with spastic equinovarus, our findings evidenced a mean TP muscle depth of 26.5 mm and a mean TP muscle thickness of 10.1 mm. Furthermore, in our patients, we observed a median spastic TP muscle echo intensity of 3 on the Heckmatt scale [23,24,25].

Patients with UMNS are disabled by paresis (impaired voluntary recruitment of skeletal motor units), contracture of soft tissues, and muscle overactivity (reduced ability to relax muscles) [26,27]. Muscle contracture includes atrophy (loss of muscle mass), shortening (loss of sarcomeres), myotendinous junction modifications, increased intramuscular connective tissue and fat infiltration [26]. Progressive changes in the spastic muscle may account for the loss of response to BoNT-A treatment [24], which is considered a first-line treatment for patients with focal spasticity [7,28]. 

In routine practice, needle insertion into the spastic TP muscle is usually performed on the base of anatomic surface landmarks [13,22]. Three methods are used for placing the needle into the TP muscle: the anterior, medial and posterior approaches [13,29]. According to previous studies based on ultrasonography evaluation of healthy volunteers, access to the TP muscle for needle insertion is safer at the tibia midpoint (midpoint of the length from the tibial tubercle to the bimalleolar line) for the posterior approach, and at the upper third of the tibia (junction between the proximal and middle thirds from the tibial tubercle to the bimalleolar line) for the anterior approach, because of the larger safety windows for needle insertion [13,18,19]. This is further confirmed by studies on cadavers, which report that the anterior approach to TP muscle is safer than the posterior one for needle insertion into the upper third of the leg [20,21]. Interestingly, the safety of insertion into the midpoint of the leg was found not to be significantly different in the safety window between the anterior and posterior approaches for needle insertion into the TP muscle of cadavers [20].

The safety window for needle insertion into a muscle is closely related to its volume [15,20,30]. A small limb volume is associated with a small safety zone [20]. Spasticity may affect limb anatomy, causing disruption of the normal muscle architecture, which can lead to a reduction of muscle thickness and an increase in muscle echo intensity [18,24,25]. Thus, in the case of a patient with spasticity, the safety window for needle insertion may be smaller than in healthy subjects or cadavers. This is in keeping with our findings about the inverse correlation found between TP muscle thickness and its depth as a consequence of muscle fibrosis due to spasticity (see also the direct association observed between TP muscle depth and its echo intensity, as well as the inverse association found between TP muscle thickness and its echo intensity) [24,25]. This is because muscle fibrosis lead to atrophy and reduction of muscle volume, with a consequent increase in the distance of the TP muscle superficial aponeurosis from the skin. In this sense, our findings may usefully inform MNP into the TP muscle for the BoNT-A treatment of spastic equinovarus foot in chronic stroke patients. In particular, clinicians may use our observations in order to choose the needle length and guide the medial approach to TP muscle BoNT-A injection. 

This study has several limitations. First, we did not compare the ultrasonographic features of TP muscle between legs (affected vs. healthy) within the same individual. This was because the main aim of this study was not to evaluate modifications of TP muscle due to spasticity, but to give some information about its treatment with BoNT-A in daily clinical practice. Second, we did not evaluate the thickness of subcutaneous tissue, or soleus and flexor digitorum longus muscles, which rely upon the TP muscle. Third, we did not perform ultrasonographic evaluation of other BoNT-A injection site for the anterior and posterior approach to TP muscle. Fourth, we did not compare the accuracy and the effects of BoNT-A injection into the TP performed with MNP, electrical stimulation and ultrasonography guidance. Fifth, no sonoelastographic evaluation of spastic TP muscle tissue elasticity was done [31]. 

To further validate our findings, larger-scale studies have to be carried out, taking into account the several limitations of this study reported above. In particular, future anatomical studies should aim to compare affected vs. healthy lower limbs in order to evaluate modifications of spastic TP muscle over time. Another future area of focus would be ultrasonographic evaluation of BoNT-A injection site for the medial approach to TP muscle in adult patients with spastic equinovarus foot due to other etiologies, considering that the same pattern of spasticity has been found to have features that differ according to its cause [32].

## 4. Materials and Methods 

This was a multicenter observational study. Inclusion criteria were as follows: age greater than 18 years; spastic equinovarus foot resulting from chronic stroke with muscle tone ≥2 on the Ashworth Scale (AS) [33]; time since the onset of stroke ≥ 6 months; time since last BoNT-A treatment ≥6 months. Exclusion criteria were as follows: involvement in other trials; intake of systemic pharmacological treatment for spasticity; presence of fixed contractures (muscle tone = 4 on the AS); presence of bony deformities at the affected lower limb; previous treatment of spastic equinovarus foot with neurolytic procedures; previous surgical treatment of spastic equinovarus foot; other neurological conditions involving the affected lower limb; other orthopedic conditions at the affected lower limb. All participants were outpatients, and gave their written informed consent for participation in the study, which was carried out according to the Declaration of Helsinki and approved by the local Ethics Committees.

All patients performed real-time B-mode ultrasonography by means of a MyLab 70 XVision system (Esaote SpA, Genoa, Italy) interfaced with a linear probe (scanning frequency 13 MHz), which was positioned in the transverse view over the marked injection site (see below), and perpendicular to the affected lower limb surface. We placed the probe gently over the skin using water-soluble transmission gel in order to avoid pressure alterations of the muscle tissue. Surface identification of the TP muscle was performed according to the Fheodoroff and Schurch’s atlas suggestions [27]. The site of injection was marked at about 50% of the distance from the medial femoral condyle to the medial malleolus, behind the posterior border of the tibia [27]. The following ultrasonographic features were evaluated: TP muscle depth (distance from the skin to superficial aponeurosis), TP muscle thickness (distance from the superficial to deep aponeurosis) and TP muscle echo intensity, which was graded on the Heckmatt scale (grade 1 = normal; grade 2 = increase of muscle echo intensity; grade 3 = marked increase of muscle echo intensity; grade 4 = very high muscle echo intensity) [23,24,25]. The whole procedure was carried out with patients in the supine position and legs outstretched.

Statistical analysis was carried out by means of the Statistical Package for Social Science for Macintosh, version 20.0 (IBM SPSS Inc., Armonk, NY, USA). Descriptive statistics were used for all the items considered. The Spearman rank correlation test was performed to assess the association between the ultrasonographic and clinical features of the spastic TP muscle. The alpha level for significance was set at *p* < 0.05. 

## Figures and Tables

**Table 1:** Patients’ demographic and clinical features.

Age (years) *mean* (*SD*)	62.3 (10.7)
Gender *male/female*	32/14
Time since stroke onset (years) *mean* (*SD*)	5.3 (3.6)
Equinovarus spasticity (AS 0–4) *median* (*IQR*)	3.0 (2.0; 3.0)
Tibialis posterior muscle depth (mm) *mean* (*SD*)	26.5 (5.0)
Tibialis posterior muscle thickness (mm) *mean* (*SD*)	10.1 (2.8)
Tibialis posterior muscle echo intensity (Heckmatt grade) *median* (*IQR*)	3.0 (2.0; 3.0)

Abbreviations: *SD*, standard deviation; AS, Ashworth scale; *mm*, millimeters; *IQR*, InterQuartile Range.

**Table 2:** Correlation matrix for study variables (Spearman ρ).

PARAMETER	Age	Time Since Onset	Equinovarus Spasticity (AS)	TP Muscle Depth	TP Muscle Thickness	TP Muscle Echo Intensity
Age	1.000					
Time since onset	0.533	1.000				
Equinovarus foot spasticity (AS)	0.778	0.067	1.000			
TP muscle depth	0.808	0.184	0.573	1.000		
TP muscle thickness	0.610	0.131	0.952	−0.541 *	1.000	
TP muscle echo intensity	0.671	0.672	0.651	0.416 *	−0.403 *	1.000

Abbreviations: AS, Ashworth Scale; TP, Tibialis Posterior. * Significant correlation (*p* < 0.05).
